# Cardiovascular magnetic resonance image analysis and mechanism study for the changes after treatments for primary microvascular angina pectoris

**DOI:** 10.1097/MD.0000000000026038

**Published:** 2021-05-28

**Authors:** Qi Huang, Wen ting Wang, Shi sheng Wang, De an Pei, Xiang qian Sui

**Affiliations:** Zhejiang Hospital of Integrated Traditional Chinese and Western Medicine (HangZhou Red Cross Hospital), Hangzhou, Zhejiang, China.

**Keywords:** cardiovascular magnetic resonance, homocysteine, nicorandil, primary microvascular angina, von Willebrand factor

## Abstract

Most cases of primary microvascular angina pectoris (PMVA) are diagnosed clinically, but the etiology and pathological mechanisms are unknown. The effect of routine clinical medications is minimal, and PMVA can progress to serious cardiovascular events. To improve the diagnosis and effective treatment of this disease, this study was designed to diagnose PMVA via cardiovascular magnetic resonance (CMR) and the coronary angiography thrombolysis in myocardial infarction (TIMI) blood flow grade, as well as to analyze vascular endothelial function to elucidate the pathogenesis of PMVA and compare the effects of routine clinical medications.

The present randomized controlled trial including a parallel control group will be conducted on 63 PMVA patients in our cardiovascular department. The patients will be selected and randomly divided into the control, diltiazem, and nicorandil groups. The control group will be administered routine drug treatments (aspirin, atorvastatin, betaloc ZOK, perindopril, and isosorbidemononitrate sustained-release tablets). The diltiazem group will be additionally treated with 90 mg qd diltiazem sustained-release capsules. The nicorandil group was additionally given 5 mg tid nicorandil tablets. Coronary angiography will be performed before treatment, the severity and frequency of chest pain will be evaluated before and after 9 months of treatment, and homocysteine and von Willebrand factor levels will be measured. Electrocardiography, echocardiography, dynamic electrocardiography, a treadmill exercise test, and CMR will be performed. Sex, age, body mass index, complications, smoking, and family history will also be recorded. The SPSS19.0 statistical software package will be used to analyze the data. The measurements will be expressed as the mean ± standard deviation. Measurement data will be compared between the groups using Student's *t*-test. A relative number description will be used for the counting data, and the chi-squaretest will be used to compare the groups. A multivariate logistic regression analysis will be performed A *P*-value < .05 will be considered significant.

The direct indices (CMR and coronary angiographic TIMI blood flow grade) may improve after adding diltiazem or nicorandil during routine drug treatments (such as aspirin, statins, and nitrates) in PMVA patients, and indirect indices (homocysteine and von Willebrand factor levels) may be reduced.

**Trial registration:**

Chinese Clinical Trial Registry (http://www.chictr.org.cn/showprojen.aspx?proj=41894), No. CHiCTR1900025319, Registered on August 23, 2019; pre initiation.

## 1 Introduction

Most cases coronary microvascular disease (CMVD) cases are diagnosed clinically, but making a definite diagnosis of CMVD remains difficult. The diagnosis can only be established by complex non-invasive examinations, such as cardiovascular magnetic resonance (CMR) or traumatic examinations (such as coronary angiography). The etiology and pathological mechanisms of CMVD are unknown;^[1–3]^ therefore, they cannot be targeted for treatment. The effect of routine clinical medication is minimal, and CMVD can progress into serious cardiovascular events.

Accordingly, CMVD has been classified into four main types based on the clinical setting in which it occurs:^[4–7]^

1. CMVD in the absence of myocardial diseases or obstructive coronary artery disease (CAD),
2. CMVD in myocardial diseases,
3. CMVD in obstructive CAD, and
4. iatrogenic CMVD. CMVD without obstructive coronary artery disease is also known as primary microvascular angina (PMVA).

This study aimed to diagnose PMVA using CMR and the coronary angiography thrombolysis in myocardial infarction (TIMI) blood flow grade and to analyze vascular endothelial function to elucidate the pathogenesis of PMVA. We expect to observe changes in the clinical symptoms, assessed using direct (CMR and coronary angiography TIMI blood flow grade) and indirect indicators (homocysteine (Hcy) and von Willebrand factor (vWF)), after effective drug treatments, which will help further elucidate the pathological mechanism and treatments for PMVA. It is hoped that the etiology, pathological mechanism, effective therapeutic drugs, and mechanism of PMVA will be established in the future based on this study.

Coronary microvasculature plays an important role in myocardial blood supply, and its dysfunction may lead to different degrees of coronary microvascular disease (CMVD).^[8]^ CMVD refers to the structure and/or function of the anterior small coronary arterioles and small coronary arterioles under the influence of various pathogenic factors. However, most diagnoses are limited to clinical experience and inference.

The coronary artery consists of three segments, including the subepicardial coronary artery with a diameter of 0.5–5 mm, the main function of which is to transport blood; the anterior small artery with a diameter of 0.1–0.5 mm, the main function of which is to stabilize pressure in the small coronary artery by vasodilation; and the small coronary artery with a diameter of < 0.1 mm, the main function of which is to regulate the tension in the blood vessel and blood flow according to myocardial metabolism. Anterior small coronary arterioles and small coronary arterioles constitute the coronary microvessels.^[9]^ The coronary microvasculature (vessels < 0.5 mm in diameter) cannot be imaged directly in vivo,^[10,11]^ but several invasive and noninvasive techniques, each with their relative advantages and disadvantages, are used to assess parameters that are directly dependent on coronary microvascular function.^[12]^ They are the main resistance vessel bed of the coronary artery and the site of myocardial metabolism.^[13,14]^

PMVA patients have typical or atypical angina pectoris symptoms (particularly exertional angina pectoris).^[15,16]^ Evidence of myocardial ischemia can be found on an electrocardiogram. The results of treadmill exercise tests are mostly positive, and the results of coronary angiography are generally normal.^[17]^ Although these small coronary arteries cannot be visualized by coronary angiography, they are the decisive factors in coronary artery resistance and blood perfusion.^[10,11]^ Among patients who undergo coronary angiography due to chest pain, 20–30% have non-obstructive lesions.^[18]^ PMVA patients have repeated attacks of exertional angina pectoris, which does not present clinically.^[19]^ Targeted drugs, such as aspirin, statins, beta-blockers, angiotensin-converting enzyme inhibitors, nitrate esters, and other conventional drugs, are often administered, but their effects are suboptimal.^[20,21]^ As conventional drug treatments are relatively ineffective, repeated medical treatments are necessary.^[22,23]^ Overuse increases the psychological and economic burdens and decreases the quality of life of patients. PMVA can also progress to serious cardiovascular events.^[19]^ Studies have reported that the risks of major cardiovascular events and all-cause mortality are significantly higher in these patients than in a healthy control group.^[24]^ Therefore, early detection and effective treatment of PMVA are very important.

The etiology and pathogenesis of PMVA are not fully understood due to the complex and multifactorial nature of the disease, which can be influenced by atherosclerosis, dysfunction of coronary microvascular endothelial cells, coronary microvascular remodeling, the inflammatory response, oxidative stress, insulin resistance, abnormal lipid metabolism, and vascular endothelial dysfunction.^[25]^ The initiating mechanism of various vascular diseases is a major hurdle to research. The vascular endothelium not only is a defensive barrier but is also implicated in endocrine and metabolic functions, mainly synthesizing and secreting substances that affect vasoconstriction. Inflammatory responses and factors are often related to endothelial injury. Inflammatory factors promote vascular endothelial proliferation and microvascular injury, which result in endothelial dysfunction.^[26]^ In turn, the intima of the blood vessel thickens, the smooth muscle of the myocardial blood vessel becomes fibrotic, and the blood vessel remodels, thereby affecting the vasomotor function of the blood vessel and further developing into microvascular structural sclerosis and abnormal blood flow regulation.

The main invasive examination for evaluating coronary microvascular function is coronary angiography. It is used mainly to evaluate perfusion of the visible epicardial coronary artery. The speed of blood flow reflects the level of microvascular resistance from the side where the epicardial coronary artery has not narrowed significantly.^[19]^

The main non-invasive technique for evaluating coronary microvascular function is CMR,^[10]^ which has the advantages of a large field of vision, multi-directional and multi-parameter imaging, high repeatability, high tissue resolution, and nonionizing radiation. The multi-output CMR scan provides information on cardiac morphology, function, wall motion, myocardial perfusion and metabolism, and myocardial activity. CMR is the gold standard for non-invasive evaluation of cardiac structure and function. CMR is used to observe cardiac structure and function directly and to determine the function of the myocardial microcirculation via the uptake of a contrast agent at different myocardial stages.^[27]^ This technique is used to evaluate any microvascular obstructions according to changes in signal intensity caused by intravenous injection of a gadolinium-containing contrast agent. Gadolinium contrast agents are extracellular contrast agents that diffuse quickly through the capillary wall into the interstitial space after entering the blood pool, but normal cellular uptake is prevented by the cell membrane. The contrast agent in the extracellular space is quickly cleared with a prolonged perfusion time, and most of the contrast is obtained by delayed scanning. The agent is discharged from the interstitial space, so the normal myocardium does not show delayed enhancement. These characteristics of CMR technology make visualization of the microcirculation one of the most promising techniques for evaluating microcirculatory disorders.^[28]^

Diltiazem is a non-dihydropyridine calcium antagonist that dilates blood vessels and prolongs atrioventricular nodal conduction by inhibiting calcium ion flow to peripheral blood vessels, coronary artery smooth muscle, and atrioventricular node cells. It reduces blood pressure, heart rate, and cardiac afterload and increases coronary artery blood flow. It is used to treat hypertension and angina pectoris, but it has a minimal effect on coronary microvascular angina pectoris.

Nicorandil has recently been used to treat PMVA, but its effect and mode of action are not understood. The following potential mechanisms have been proposed.^[29]^ Nicorandil opens ATP-sensitive potassium channels and reduces Ca^2+^ influx by increasing intracellular K^+^ outflow, resulting in the relaxation of vascular smooth muscle, coronary arterioles, and obstruction. Therefore, greater vasodilation improves microcirculation.^[30]^ Nicorandil also has nitrate ester side chains with nitrate-like effects. Nicorandil increases the synthesis of endogenous nitric oxide, dilates subepicardial coronary arteries and arterioles, increases coronary blood flow, expands systemic arterioles and volumetric vessels, reduces peripheral resistance, reduces the pre- and post-load of the heart and myocardial oxygen consumption, and increases collateral circulation and subendocardial blood supply.^[31]^ Nicorandil inhibits the release of inflammatory factors and the subsequent inflammatory response, reduces endothelial cell apoptosis, and improves vascular endothelial function.^[30]^ (4) An elevated blood lipid level is an important factor in atherosclerosis.^[30]^ Nicorandil prevents lipid deposition in the arterial walls and cells and has anti-atherosclerotic effects.^[32]^ Nicorandil is the preferred drug for treating PMVA.^[20,33]^

Hcy is an intermediate product of methionine metabolism that reflects the metabolic status of the vascular endothelium and is an independent risk factor for cardiovascular diseases.

vWF is a polymer glycoprotein secreted by vascular endothelial cells that plays a key role in platelet aggregation by mediating adhesion between platelets and vascular endothelial cells and is a specific marker of vascular endothelial cell damage.

The objectives of this study are to evaluate the changes in CMR images of PMVA patients before and after different drug treatments and to explore the possible pathogenesis of PMVA by determining vascular endothelial function in patients with PMVA.

The purpose of this study is to investigate the pathogenesis of PMVA and identify potential therapeutics. Diltiazem and nicorandil will be administered based on routine drug therapies (aspirin, statins, and nitrates). The main index measured will be CMR imaging. Secondary indicators will be improvements in clinical symptoms and changes in Hcy and vWF levels after treatments. Subsequently, the potential pathogenesis of PMVA and the specific efficacy and mechanism of effective drugs for PMVA treatment will be analyzed.

## 2 Methods

### 2.1 Registration

**Figure 1:**
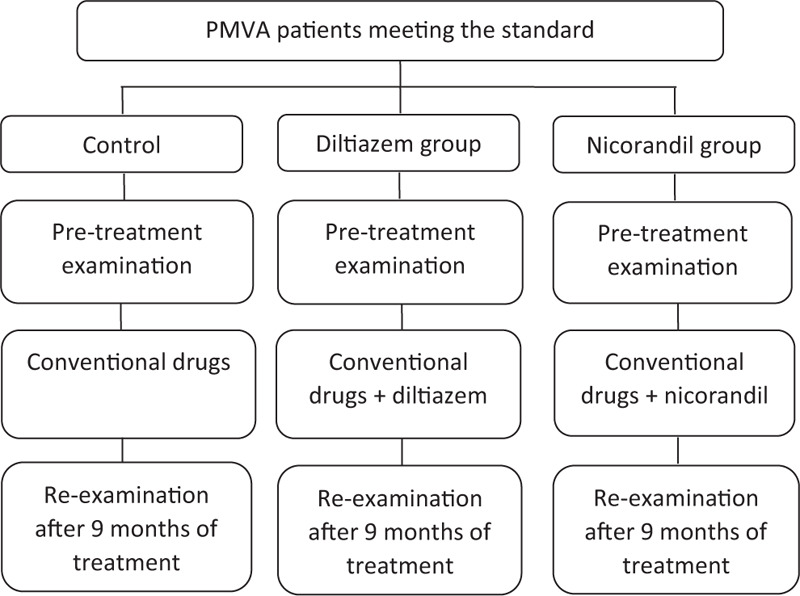
The pre-treatment examination includes an assessment of the degree and frequency of chest pain, blood sampling, ECG, DCG, echocardiography, a treadmill exercise test, CMR, and coronary angiography.

This trial had been registered before recruitment on the Chinese Clinical Trial Registry (http://www.chictr.org.cn/showprojen.aspx?proj=41894 No.ChiCTR1900025319). Does not appear in this manuscript but can be found in trial registry record. We will conduct the principles of the Declaration of Helsinki (2004 version) during the trial. The study protocol also has been approved by the Ethics Committee of Hangzhou Red Cross Hospital before recruitment (Approval number: 2018–09). This study is a randomized, controlled, Single-blind trial. The study flowchart was shown in Figure 1.

Personal contact information will be accessible only to the research team members. All personal patient information will be protected. All information will be recorded using codes assigned to the patients (and without mentioning patients’ names). We do not anticipate any harm as a result of participation in this study. The results of this study will be disseminated in journals. The present study protocol was prepared following the SPIRIT statement. If important modifications are made, the Committee of Ethics of Hangzhou Red Cross Hospital will be informed, and new protocols will be uploaded to the CHiCTR.

### 2.2 Recruitment

The present randomized controlled trial with a parallel control group will be conducted on 63 patients in our cardiovascular department. All patients will meet the inclusion criteria and agree to participate in the study. This protocol is organized based on the Standard Protocol Items: Recommendations for Intervention Trials (SPIRIT) guidelines, which provide the Standard Protocol Items. The SPIRIT checklist for the present study is provided as an Additional file.

Zhejiang Integrated Traditional and Western Medicine Hospital (HangZhou Red Cross Hospital) is located at 208 Huancheng East Road, Hangzhou. Hangzhou is the provincial capital of Zhejiang Province, China.

The first author will explain the research objectives and methods including its purpose, processing, scheduling, and possible risks and benefits to the participants during the inclusion criteria meeting before enrollment. The first author will obtain written consent from the patients willing to participate in the trial. No ancillary studies are planned.

### 2.3 Inclusion criteria

The inclusion criteria will be age 18–75 years and conformation to the following diagnostic criteria:

1. typical exertional angina pectoris for at least 6 months,
2. conventional 12-lead ECG recording and ST-segment offset measurement at 80 ms after the J-point,
3. positive treadmill exercise test [ST segment ischemic descent (>0.01 mm)] or dynamic electrocardiography monitoring showing at least one ST-segment ischemic descent (>0.01 mm),
4. normal echocardiographic left ventricular function and > 55% left ventricular ejection fraction,
5. coronary artery angiography showing normal or almost normal results (normal or irregular epicardial coronary artery wall, degree of stenosis < 20%), no spontaneous coronary artery spasm, and TIMI blood flow grade 1–2, and
6. coronary microcirculation disorder confirmed by CMR.

### 2.4 Exclusion criteria

The exclusion criteria will be

1. organic heart disease, including coronary artery spasm, coronary artery bridge, primary cardiomyopathy, congenital heart disease, valvular heart disease, hypertrophic cardiomyopathy, pulmonary heart disease, or hypertensive heart disease;
2. myocardial injury, including myocarditis and systemic combined myocardial injury;
3. history of myocardial infarction, coronary intervention, or coronary artery bypass grafting;
4. arrhythmias requiring drug control;
5. complete left bundle branch block, degree II and III atrioventricular block, or sick sinus syndrome;
6. severe congestive heart failure;
7. a malignant neoplasm;
8. lung, esophageal, or aortic dissection or other diseases causing chest pain;
9. severe liver or kidney dysfunction;
10. acute or chronic inflammatory diseases and severe trauma;
11. endocrine system diseases;
12. anemia;
13. peripheral vascular diseases;
14. active biliary and hepatobiliary diseases;
15. contraindication by magnetic resonance examination;
16. inability to take medication; and
17. pregnant or lactating women.

### 2.5 Sample size estimation

Excel software will be used for data input. The chi-square test of multiple proportions will be used to calculate the sample size. The effect size of the chi-square test was.4, the significance level (alpha) was.05, the power of the test was.8, and the number of degrees of freedom was 2. A sample size of 63, or 21 in each group, was calculated based on these parameters.^[34]^

### 2.6 Study drug

63 patients with PMVA will be selected and randomly divided into the control, diltiazem, and nicorandil groups. The control group will be given routine drug treatment (aspirin enteric-coated tablets [100 mg Bayer Medical Health Co., Ltd., Chinese medicine quasi-word J20130078] orally once at night, atorvastatin calcium tablets [20 mg; Pfizer Pharmaceuticals Co., Ltd., Chinese medicine quasi-word J20030048] orally once at night, isosorbide mononitrate sustained-release tablets [40 mg; Lunan Beit Pharmaceutical Co., Ltd., Chinese medicine quasi-word H19991039] orally once daily, betaloc ZOK [47.5 mg; AstraZeneca Chinese medicine quasi-word J20100098] orally once daily, and perindopril tablets [4 mg; Servier Tianjin Pharmaceutical Co., Ltd., Chinese medicine quasi-word H20034053] orally once daily. The diltiazem group will be given the routine drug treatment plus diltiazem sustained-release capsules (90 mg; Shanghai Aifa Pharmaceutical Co., Ltd., Chinese medicine quasi-word H20020711) orally once daily. The nicorandil group will be treated with the routine drug treatment plus nicorandil tablets (5 mg; Sino Foreign Pharmaceutical Co., Ltd., Chinese medicine quasi-word H20150023) orally three times daily.

### 2.7 Randomization and blinding

A person not involved in the study will use the simple random technique to determine the allocation sequence with a 1:1:1 ratio. To ensure a concealed allocation, this person will write the type of intervention based on a predetermined sequence and place it in an opaque envelope. These envelopes will be encoded. To observe blinding during data collection, a coworker will perform the intervention plan, and another coworker blinded to the intervention will collect the data. We do not predict any circumstances in which unblinding will be necessary.

Once the results are gathered and the data are imported into SPSS19.0 software, the researcher performing the data analysis will be blinded to the type of intervention used for each group.

### 2.8 Interventions

We will explain to each volunteer the risk and benefit of this trial at the first visit (-t1). We will reassure the volunteers about the confidentiality of their information, answer their questions regarding the study, and explain the results at the end of the study. Pre-treatment (t0) examinations will include an assessment of the degree and frequency of chest pain, blood tests, electrocardiography, dynamic electrocardiography, echocardiography, a treadmill exercise test, CMR, and coronary angiography. The medications will be administered during t1–t9, and evaluations will include an assessment of chest pain and frequency, blood tests, electrocardiography, dynamic electrocardiography, echocardiography, a treadmill exercise test, and CMR. Blood tests will include measurements of Hcy and vWF levels at the end of the trial (t9). Sex, age, body mass index, complications, smoking, and family history will be recorded. The ethics committee will monitor the entire procedure, including data collection. We do not plan to conduct an interim analysis.

Participation in this study will not have a major impact on the daily life of the patients, and coronary angiography will be performed in our hospital. Coronary angiography is a minimally invasive procedure, and the relevant surgical risks will be specified during the preoperative conversation. Contraindication to the resonance examination is the only limitation of CMR. Blood analyses, electrocardiography, echocardiography, the treadmill exercise test, and dynamic electrocardiography will be performed safely. The drugs used in the study have a low incidence of side effects, such as allergy, digestive discomfort, or abnormal liver and kidney function. These drugs have been widely used in clinical practice for many years and have been demonstrated to be safe and effective. Regular inspections are important, and treatments will be discontinued when serious complications occur.

**Figure 2:**
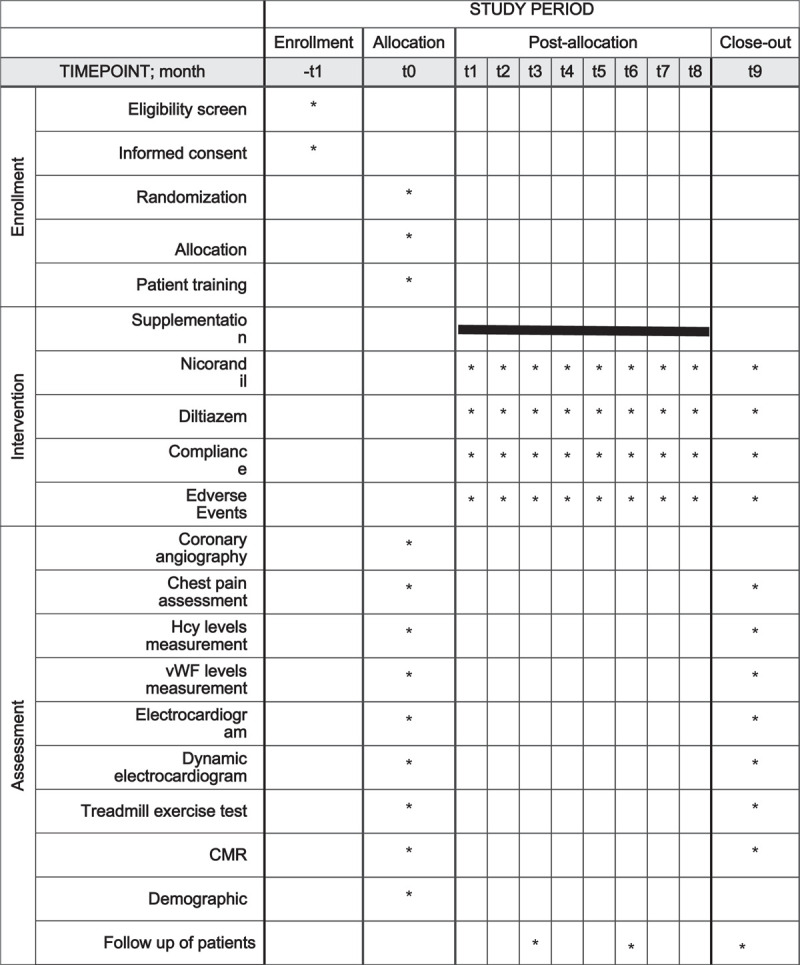
Schedule of enrollment, intervention, and assessments based on the SPIRIT guidelines. SPIRIT, Standard Protocol Items, Recommendations for Interventional Trials; Demographic variables, including sex, age, body mass index, complications, smoking, and family history were collected.

Patients will be followed up every 3 months (t3 and t6) in the outpatient clinic. This frequency will be increased if necessary. If drug side effects occur, the study will be terminated immediately, and treatment will be started. Relevant concomitant care and the interventions permitted during the trial are shown in Figure 2.

#### 2.8.1 Coronary angiography method

Judkin's method will be used for coronary angiography to evaluate the blood flow velocity in the epicardial coronary artery. At present, the most commonly used evaluation index is thrombolysis in myocardial infarction (TIMI classification). Specific TIMI criteria are as follows: 0, no forward blood flow beyond an occlusion;

1, slight, weak forward blood flow beyond the occlusion but no distal vascular bed filling;
2, blood flow reaches the distal vascular bed, but the forward blood flow is slow or delayed;
3, blood fills the distal vascular bed, and the forward blood flow is normal.

#### 2.8.2 MR myocardial perfusion method

The first perfusion and late gadolinium enhancement (LGE) CMR scans will be performed using the German Siemens 1.5T superconducting magnetic resonance scanner (MagnetomAvanto, Siemens) with a gadolinium contrast agent.

#### 2.8.3 MR myocardial perfusion data analyses

Segmental perfusion and LGE will be analyzed semi-quantitatively. A segmental perfusion analysis is a visual analysis of each myocardial segment. According to the 17-segment method of the American Heart Association, the wall motion of each segment will be classified as grade 1 (normal movement), grade 2 (decreased movement), grade 3 (transportation), or grade 4 (dyskinesia). The LGE analysis will use a 4-point scale (0 = absolutely normal, 1 = may be normal, 2 = may be pathological, and 3 = pathological). Scores of 2 or 3 points will be considered abnormal. The image evaluation will be blindly conducted by two experienced radiologists, and consensus will be used as the criterion.

The exercise load test will be performed in the morning using a treadmill tester. The exercise treadmill test will be performed using the MAX-1 treadmill (Marquette Electronics Inc., Marquette, MI, USA) with sub-maximum load electrocardiography and the Bruce scheme. A 12-lead electrocardiogram will be recorded before, during, and after the exercise. The criteria for judging positive results will be the appearance of typical angina pectoris and the level of oblique ischemic depression of the ST segment during and after exercise 80 ms after the J-point is > 1.0 mm and lasting at least 1 min. If one of the following conditions occurs, the test will be terminated:

1. typical angina attack, severe dizziness, sweating, severe asthma, pale skin, extreme fatigue, dyskinesia, and other symptoms,
2. ST-segment depression (> 0.2 mV or T-wave inversion),
3. severe arrhythmia (ventricular tachycardia, atrial fibrillation, multiple frequent ventricular premature beats, atrioventricular transmission),
4. heart rate decrease to 25 beats/min or increase to > 85% of the maximum target heart rate,
5. blood pressure increase to 220/110 mmHg or systolic blood pressure decrease to >15 mmHg, and
6. subject asks to stop.

Treatment will be considered effective if the ST-segment depression recovers > 0.05 mV, wave inversion becomes shallower than 25%, or the T-wave becomes flat and upright. Treatment will be considered ineffective if the ECG does not change significantly.

The curative effects will be divided into marked, effective, and ineffective levels according to the angina symptom relief criterion. A marked effect will be classified as the disappearance of angina pectoris, > 80% reduction in the number of attacks, or decrease in the isosorbide nitrate dosage by > 80% after one course of treatment. Effective treatment will be classified as an improvement in angina pectoris or a 50–80% reduction in the number of attacks after one course of treatment. Ineffective treatment will be classified as a < 50% reduction in the number of attacks or the dosage of isosorbide nitrate after one course of treatment. The total effective rate will be calculated using the following formula: (significant + effective) / total number of cases × 100%.

#### 2.8.4 Hcy determination

Venous blood (3 ml) will be collected and placed in a blank glass tube to analyze hemorrhagic clearance. The blood will be stored at −70°C. The Hcy ELISA kit (ADI/Dellwin Co.) will be used for the Hcy analysis according to the manufacturer's protocol.

#### 2.8.5 VWF determination

Venous blood (3 ml) will be collected and placed in a plastic test tube containing a 1:10 volume ratio of 0.109 mmol/L sodium citrate anticoagulant. The blood will be separated by centrifugation at 3000 r/min for 10 min and stored at −70°C for testing. The vWF ELISA kit (ADI/Dellwin Co.) will be used for the vWF analysis according to the manufacturer's protocol.

## 3 Data collection and management

### 3.1 Evaluation

#### 3.1.1 Primary outcome

(1) Improvements in the results of CMR imaging in PMVA patients during routine drug treatment (i.e., aspirin, statins, and nitrates) and reductions in the indirect indices (Hcy and vWF levels) in these patients after adding the diltiazem or nicorandil treatments.
(2) A significant improvement in the nicorandil group.
(3) Determining the specific efficacy and possible mechanism of these drugs to help further understand PMVA.

#### 3.1.2 Second outcome

(1) Hcy and vWF levels will be measured in patients with PMVA diagnosed by clinical symptoms, electrocardiography, CMR, and coronary angiography, and these indices reflecting vascular endothelial function will be significantly increased in PMVA patients.
(2) In comparison with regular treatment strategies, including aspirin, statins, beta-blockers, angiotensin-converting enzyme inhibitors, and nitrates, the clinical symptoms and results of electrocardiography and treadmill exercise tests of PMVA patients may improve after adding the diltiazem or nicorandil treatments.

#### 3.1.3 Adverse events

All adverse events will be documented during the intervention. If any adverse event occurs, it will be immediately reported and the participant will be received the corresponding treatment. We will analyze the causality to determine the severity and the relationship between the adverse events. Serious adverse events determined to be drug-induced will be reported to the ethical committee timely and discuss whether we should discontinue or modify the criteria.

The patients will be contacted by telephone every 3 months to inquire about their current condition and treatment, as well as whether they have any complications. The patients will be allowed to present to the hospital for further consultation, if necessary, to promote the participant's retention in the study and complete follow-up. The completed clinical outcome data of participants who discontinue or deviate from the protocols will be collected.

#### 3.1.4 Patient and public involvement

Neither the patients nor the public will be directly involved in the development of this study protocol. We will disseminate the results to the study participants through journal publications and research conferences.

#### 3.1.5 Statistical analysis

SPSS 19.0 software will be used to analyze the data. The normality of the data distribution will be investigated, and an appropriate transformation will be used if the distribution is not normal. Measurements will be expressed as mean ± standard deviation (SD). Student's *t*-test will be used to compare the measurement data. A relative number description will be used for the counting data, and the chi-square test will be used for intergroup comparisons. Multivariate logistic regression analysis will be performed. A *P*-value < .05 will be considered significant.

## 4 Discussion

The incidence of chronic diseases, such as hypertension, hyperlipidemia, hyperuricemia, and diabetes mellitus has increased significantly with the aging of society, and onset is occurring at a younger age. The proportion of smokers in the general population remains high. Environmental changes and other factors have increased the occurrence of chronic inflammation, leading to atherosclerosis and impaired vascular endothelial function, which may result in PMVA.^[35]^

As the structure and function of coronary microvessels are different from those of subepicardial coronary arteries, the traditional effective drugs for coronary heart disease cannot be extrapolated for use in patients with PMVA. Nitrates effectively relieve angina pectoris symptoms of coronary heart disease, and medications can be used to stabilize the condition and effectively delay the progression of coronary heart disease. Drugs such as aspirin, statins, and beta-blockers have little effect on PMVA.

PMVA patients lack effective treatments, but their risk of major cardiovascular events and all-cause mortality are significantly higher than those of healthy individuals.^[20]^ Due to the poor effect of routine drug treatment, PMVA patients repeatedly visit clinics and undergo numerous examinations and multiple rounds of treatment, which has increased the economic burden on patients and society. Even after routine drug treatment, PMVA can still develop into a serious cardiovascular event with dire consequences. If the etiology and pathogenesis of PMVA were known, PMVA could be applied for more effective therapeutics. It is expected to be popularized throughout the country after the publication of this article, which will provide economic and social benefits. The direct indices (CMR and coronary angiographic TIMI blood flow grade) could improve, and reductions in the indirect indices (Hcy and vWF levels) may occur after adding diltiazem or nicorandil during routine drug treatments (such as aspirin, statins, and nitrates) in PMVA patients.^[35]^

### 4.1 Trial status

The protocol's version number and date are V1.0 and August 23, 2019. Recruitment will begin on June 3, 2021, and data collection will probably be completed on December 31, 2022. The data will be updated in the Chinese Clinical Trial Registry.

## Acknowledgments

We thank all care team members and lab researchers for their dedicated participation in this project; I would like to express my gratitude to all those who helped me during the writing of this thesis and for the support of my family members.

## Author contributions

**Conceptualization:** Qi Huang.

**Data curation:** Qi Huang, De an Pei, Xiang qian Sui.

**Formal analysis:** Qi Huang.

**Funding acquisition:** Qi Huang.

**Investigation:** Qi Huang, Wen ting Wang, Shi sheng Wang.

**Methodology:** Qi Huang.

**Project administration:** Qi Huang.

**Resources:** Qi Huang.

**Supervision:** Qi Huang.

**Visualization:** Qi Huang.

**Writing – original draft:** Qi Huang.

**Writing – review & editing:** Qi Huang.
